# Management of Fractures of the Thoracolumbar Spine―A Narrative Review

**DOI:** 10.3390/jcm15031008

**Published:** 2026-01-27

**Authors:** Sven Y. Vetter, Andreas Badke, Sandra Buchmann, Stefan Hauck, Peter Heumann, Frank Kandziora, Philipp Kobbe, Sebastian Krüger, Christiane Kruppa, Bernhard W. Ullrich, Philipp Schleicher

**Affiliations:** 1 Department for Orthopaedics and Trauma Surgery, Heidelberg University, BG Klinik Ludwigshafen, 67071 Ludwigshafen, Germany; 2 Center for Spinal Surgery and Spinal Cord Injury, BG Kliniken Ludwigshafen und Tübingen gGmbH, BG Klinik Tübingen, 72076 Tübingen, Germany; abadke@bgu-tuebingen.de; 3 Center for Spinal and Pelvis Surgery, BG Klinikum Duisburg gGmbH, 47249 Duisburg, Germany; sandra.buchmann@bg-klinikum-duisburg.de; 4 Center for Spinal Surgery, BG Klinikum Murnau gGmbH, 82418 Murnau am Staffelsee, Germany; shauck@bgu-murnau.de; 5 BG Klinikum Unfallkrankenhaus Berlin gGmbH, 12683 Berlin, Germany; peter.heumann@ukb.de; 6 Center for Spinal Surgery and Neurosurgery, BG Unfallklinik Frankfurt am Main gGmbH, 60389 Frankfurt am Main, Germany; frank.kandziora@bgu-frankfurt.de; 7 BG Klinikum Bergmannstrost Halle gGmbH, 06112 Halle, Germany; philipp.kobbe@bergmannstrost.de (P.K.); bernhard.ullrich@bergmannstrost.de (B.W.U.); 8 University Medicine Halle, 06120 Halle, Germany; 9 Spine Center, BG Klinikum Hamburg gGmbH, 21033 Hamburg, Germany; s.krueger@bgk-hamburg.de; 10 Berufsgenossenschaftliches Universitätsklinikum Bergmannsheil gGmbH, Ruhr-Universität Bochum, 44789 Bochum, Germany; christiane.kruppa@bergmannsheil.de

**Keywords:** spine trauma, epidemiology, history, anterior approach, posterior approach, minimally invasive spine surgery

## Abstract

The thoracolumbar region affects 60 to 80% of the 4 million spine fractures occurring annually, making them a global health threat. Management has evolved from early fixation systems to minimally invasive techniques, reducing muscle trauma and recovery time. Fractures are classified into compression, distraction, and translation types, with stability guiding treatment decisions. Surgical options include open and minimally invasive procedures, each with benefits and drawbacks. The choice of treatment depends on fracture type, neurological deficits, and patient factors. Advances in technology continue to improve outcomes, but further research is needed to determine optimal management strategies.

## 1. Background and Epidemiology

Fractures of the spine remain a significant cause of morbidity and mortality worldwide. According to data from the Global Burden of Disease (GBD) Project, 4.73 million cases occurred worldwide in 2021 alone, leading to more than 369,000 years lived with disability (YLD) [1].

The thoracolumbar junction (i.e., injuries to the rigid thoracic spine and the flexible lumbar spine, typically occurring between the 11th thoracic (Th11) and second lumbar (L2) vertebral bodies) is affected in 60% to 80% of all cases. A recent analysis of 39 studies by the World Federation of Neurosurgical Societies (WFNS) suggests an annual incidence of thoracolumbar fractures of about 30/100,000 inhabitants, including osteoporotic fractures [2].

Up to 20% of injuries are associated with neurological deficits [3,4], and it is estimated that one-third of all patients suffer from long-lasting functional impairment [5,6].

The German Social Accident Insurance (DGUV) spent €540 million on the consequences of thoracolumbar fractures in 2023, of which €80 million was paid for inpatient treatment.

Understanding the anatomy is crucial for effectively diagnosing and managing fractures of the thoracolumbar region. In general, they are classified as compression (Type A), distraction (Type B), and translation fractures (Type C), according to the mechanism of impact, and resulting destruction of bone structures and the ligamentous apparatus.

Compression fractures to the thoracolumbar junction remain the most common fracture type. They occur when a vertebral body collapses or is compressed under axial loading force. Grading them as stable or unstable is significant for clinical decision-making.

The AO Spine System defines stable fractures (A0 and A1) as those that do not involve the posterior wall of the vertebral body. The rare vertebral split fracture (A2) and incomplete burst fracture (A3) types may lead to instability or spinal canal compression, making posterior stabilisation obligatory, possibly supplemented by anterior fusion. The complete burst fracture type A4 is preferably treated operatively. Type B distraction fractures due to hyperflexion or hyperextension of the thoracolumbar spine are typically unstable since the anterior and posterior columns of the vertebra are involved, making posterior stabilisation necessary. Translational type C fractures are, by definition, highly unstable and require posterior stabilisation, either alone or in combination with anterior fixation in a 360-degree fashion.

About half of all thoracolumbar fractures are currently managed surgically. The main indication for surgical treatment is to restore stability, sagittal alignment and a compromised spinal canal to relieve actual or avoid subsequent neurological symptoms and consequences.

In cases of acute neurological impairment, there is no doubt that the spinal canal must be urgently decompressed, and the surrounding protective structures relieved to either resolve deficits or prevent them.

Yet, there is an ongoing debate over whether these fractures must be surgically fixed regularly if there is no neurological compromise.

This narrative review is intended to provide a German perspective on this issue for international stakeholders, without claiming to be exhaustive. Given this type of manuscript, we combined clinical expertise and opinions with the current best scientific evidence from a selective literature search.

## 2. Evolution of Surgical Management

The dorsal approach has been the standard procedure since the early days of operative spinal fracture treatment. Based on Magerl’s external fixator, Dick and Kluger developed internal fixator systems with trans-pedicularly inserted screws in the 1980s, which were stably connected to the longitudinal rods [7].

With this technique, a sound primary reduction of the spine could be achieved with high stability and, simultaneously, the possibility of reducing the number of instrumented segments. This represented a milestone in the treatment of spine fractures.

Kuner also showed that the spinal canal could be significantly widened primarily through ligamentotaxis and, secondarily, through remodelling of the vertebral body [8].

However, these techniques do not always result in fusion of the affected vertebral bodies. Intersegmental transpedicular cancellous bone grafts were proposed to provide long-term stabilisation with anterior fusion. They aimed to replace the traumatically injured intervertebral disc with a stable bony fusion without using anterior approaches, which were still considerably invasive at the time. However, the results were disappointing, so the focus was on minimising the access trauma associated with the classic anterior approaches [9].

The first large-scale German multicenter study on stabilisation of the thoracic and lumbar spine was conducted in the early 2000s. It revealed that posteriorly instrumented patients had a significantly higher loss of correction than posteroanterior stabilised patients. Yet, during the short follow-up period, it was impossible to demonstrate the clinical relevance of this loss of correction.

At the same time, the first percutaneous systems for posterior stabilisation were introduced. Minimally invasive placement of pedicle screws became increasingly popular, and new systems were introduced to the market in short intervals. The third generation of percutaneous screw systems now available allows for sufficient fracture reduction, long-distance instrumentation, and cement augmentation [10]. The latter represents an essential innovation given the epidemiologic rise in osteoporosis prevalence.

The biomechanical significance of the anterior spine has become increasingly clear since the publication of Whitesides’ two-pillar concept [11].

Open anterior approaches to the spine, which were initially associated with substantial morbidity, were significantly developed further in the 1990s by Bühren and Beisse using thoracoscopic techniques [12]. In addition, improved retractor systems have positively influenced the comorbidity of anterior approaches.

The development of intraoperative 3D imaging and the increasing availability of computer-assisted navigation systems made minimally invasive techniques more accessible.

Minimally invasive anterior techniques are now part of the standard repertoire of the traumatological spine surgeon, and the reconstruction of the injured anterior spine is also internationally recognised as necessary [13].

This narrative review aims to provide an overview of the development of spinal surgery and to highlight the changes in spinal care over the past 25 years.

Restoring alignment in the sagittal and coronal planes is mandatory. The sagittal balance refers to the spine’s alignment in the sagittal plane, which is critical for maintaining proper posture, functional mobility, and overall spinal load bearing by minimising stress on spinal structures, including adjacent vertebrae, discs, and ligaments. Imbalances increase strain and spinal degeneration, resulting in a worse clinical outcome. Because of this, surgical techniques addressing the anterior column have evolved and are therefore much more widely used to regain physiological alignment in thoracolumbar fractures.

Surgical techniques and treatment options change over time. Especially in spinal surgery, the treatment methods have changed significantly over the last two decades. Intraoperative imaging and enabling technologies, for instance, have evolved dramatically. Additionally, preoperative and perioperative surgical planning modules influenced screw placement and column correction methods. Nonetheless, the development of instruments, retractors, and implants, especially for minimally invasive procedures, significantly influenced surgical techniques. This overview describes the development and evolution of spinal trauma care.

## 3. Therapeutic Options and Surgical Methods

The main advantage of the open freehand technique for pedicle placement is that anatomical orientation is possible. With an open posterior approach, decompression of the spinal canal and osteotomies for alignment corrections are feasible. Following instrumentation, decompression, and reduction, a posterior fusion with an autologous or allogenic bone graft can achieve bony fusion for long-term stability.

Disadvantages such as muscle trauma and higher infection rates of open procedures must be considered [14,15]. Since the beginning of the 21st century, the minimally invasive technique of pedicle screw placement using fluoroscopy has been increasingly implemented for the treatment of thoracolumbar spine fractures, as compared to the open technique. Since the risk of permanent damage to the multifidus muscles was proven to be a direct result of the open technique resulting in an impairment of more than 25% of the muscle activity [16], percutaneous procedures showed a relative reduction of that complications (9.5% vs. 18.8%). Additionally, they led to decreased intraoperative blood loss, shorter operation time, and hospitalization (7.1 vs. 12.8 days) in studies treating >1000 patients [17,18].

While placing percutaneous pedicle screws, fluoroscopy is used to locate the screw entry point. For each screw, a skin incision approximately 1 cm in length is performed, slightly lateral to the pedicle projection on anteroposterior imaging. The multifidus and longissimus muscles are dissected bluntly after opening the subcutaneous tissue and thoracolumbar fascia. Afterwards, a cannulated pedicle needle is placed and tapped in to gain access to the pedicle, then advanced through the latter while checking its correct position in the pedicle (until it reaches the medial border of the pedicle image in AP imaging) and controlling its forward movement till the posterior wall of the vertebral body (in lateral imaging). Then, the needle is advanced to the center of the vertebral body. A K-wire is introduced via the cannulated pedicle needle. After dilating the soft tissue, the canulated pedicle screw is inserted over the K-wire. These steps are repeated until all planned pedicle screws are inserted. Afterwards, a rod is placed percutaneously on every side beneath the fascia and reduced into the pedicle screws before tightly secured. Initially, implementing the percutaneous technique faced limitations regarding optimal closed reduction of compression spine fractures. In the meantime, this issue could finally be addressed by developing new surgical tools to achieve excellent results.

Nevertheless, drawbacks such as poor imaging quality remain challenging, especially in obese patients and in the upper thoracic spine, where a high risk of screw displacement is likely. Another disadvantage of the technique is that it increases radiation exposure for both the surgeon and the patient. Despite all restrictions, the percutaneous fluoroscopy-based technique plays a vital role in spine traumatology regarding its cost-effectiveness, low-level technical requirements, and safety for managing thoracolumbar spine fractures.

Spinal navigation has been established for over 25 years [19,20]. A clear advantage is improved accuracy (Navigation group: 92.66% vs. Fluoro-based group: 88.08%) of screw placement, especially in complex regions, such as the cervical and upper thoracic spine. Initially, the surgical site and the navigation system were merged using surface matching or fluoroscopy [21,22]. For 3D navigation, an intraoperative 3D scan is required, and the OR personnel may leave the control zone after the reference array is mounted to the patient [23,24,25].

When using optical tracking systems, it is necessary to respect a direct visual sight between the camera and the patient array. This restricts the options for positioning equipment and the surgical team in the operating theater [26]. It must be emphasized that visualized images on the navigation platform must be constantly revised with the real anatomy, since inaccuracies may accidentally occur [27].

Under conservative treatment, most thoracolumbar fractures may also achieve osseous healing but will not regain spinal alignment.

Primary reduction follows Hippocrates’ principles and may be applied in kyphotic A- and B-type fractures according to the AO Spine classification scheme [28]. At first, axial traction is applied to the spine, followed by manual compression under fluoroscopic control. In the next step, the reduction can be completed either in open or MIS procedures with reduction tools, monoaxial screws, and pre-bent rods [29,30].

Commonly, open reduction after sufficient decompression to avoid surgery-related neurological deterioration is necessary in type C fractures [28]. Discussing posterior-only (instrumentation vs. fusion) and two-column surgery in thoracolumbar fractures remains controversial. It depends on fracture morphology and level, neurologic symptoms, patient variables, and surgeons’ training [31].

Until now, there has been no definitive answer as to the best treatment method for thoracolumbar fractures, because of the diversity of fracture patterns, patient profiles, and surgical techniques. This leads to limited comparability across studies, institutions, and individual researchers [32]. Neither posterior short-segment (PS), posterior long-segment (PL), nor anterior combined with posterior (AP) techniques were able to maintain the corrected kyphosis angle [33,34]. Posterior fusion, in addition to internal fixation, showed no benefit in the degree of kyphosis correction, loss of kyphosis correction, or final angle of kyphosis [35]. A multicenter prospective German study reported a lower rate of kyphotic malalignment after combined posteroanterior approaches compared to posterior-only instrumentation [36]. A McCormack load-sharing score up to 6 proved to be a reliable threshold for posterior instrumentation only [37,38]. Additional anterior stabilization is recommended with an initial kyphotic deformation of 20 or more degrees and an osseous defect after restoring the anatomical alignment [39].

## 4. Posterior-Only Instrumentation

Posterior pedicle-screw instrumentation remains the standard of care for thoracolumbar fractures [40]. There is debate in the spine surgery community about the utility and value of long-segment or short-segment instrumentation with or without intermediate screws, additional fusion, etc. However, these technical features may have little if any impact on patient-centered outcomes [41]. Satisfactory clinical and radiological results were observed in both the posterior-only and anteroposterior groups, with distinct differences because of donor side pain, blood loss, duration of surgery, and hospitalization in over 400 cases [42,43,44,45]. To minimize blood loss, operative time, and hospital stay, bisegmental minimally invasive posterior instrumentation with additional kyphoplasty of the fractured vertebrae instead of anterior stabilization was introduced to treat elderly patients [44].

Some limiting factors of posterior-only instrumentation were resolved by developing new implants, novel reduction techniques, and introducing the percutaneous approach [43,45,46]. However, loss of correction and instrumentation failures remain the main limitations of posterior-only instrumentation, especially in fractures with a substantially compromised anterior column [39]. In mid-lumbar A3 fractures, posterior-only short-segment fixation did not maintain kyphosis correction at final evaluation [47].

Transpedicular screw fixation within the context of a fixateur interne system is currently considered the biomechanical gold standard, as it offers superior stability and allows for three-dimensional repositioning and targeted manipulation of each instrumented vertebra. Monoaxial screw systems, due to the rigid configuration of the screw head and its fixed connection to the longitudinal rod, impose limitations on screw placement, particularly in anatomically challenging circumstances. To address these issues, polyaxial screw heads were developed, permitting variable angulation in the linkage between the screw and the longitudinal rod and thus providing increased flexibility in screw trajectory and positioning.

This is especially advantageous in cases of fractures involving partially disrupted vertebral bodies, as it enables screw purchase in areas of intact bone. For multi-level (long-segment) instrumentation—such as in multiple-level injuries—the polyaxial design also facilitates accurate adaptation of implants to spinal geometry. However, the increased flexibility of polyaxial screw heads comes at the cost of reduced angular stability when locked, which can lead to a higher rate of loss of reduction during short-segment constructs. Anterior column support (e.g., cage implantation) or the use of long-segment instrumentation can be employed to counteract this deficit in stability.

## 5. Two-Column Surgery

**Figure 1:**
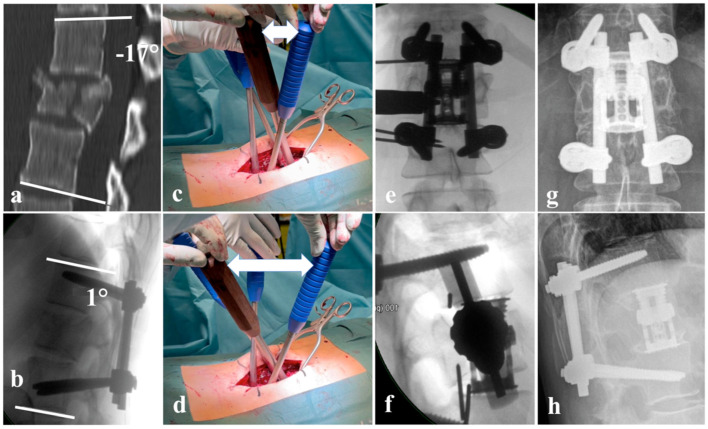
(**a**) A4, N0 fracture of L1 according to AOSpine classification with relative spinal canal narrowing and 17° fracture-related kyphosis; (**b**) lateral intraoperative X-ray after fracture reduction to 1° lordosis; (**c**) position of the reduction levers before lordotic reduction; (**d**) position of the reduction levers after reduction; the white double arrow marks the increase in distance from (**c**–**f**) intraoperative X-ray showing the positioning of the distractible vertebral body replacement cage; (**g**,**h**) 15-year follow-up with unchanged position of the fused segments.

**Figure 2:**
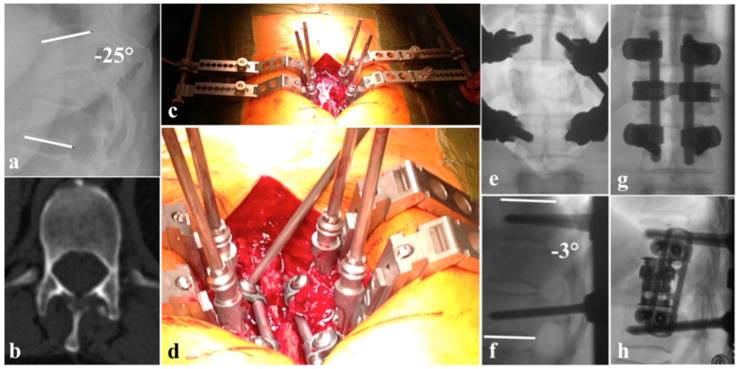
(**a**) Kyphotic deformation due to B2 Th10/11 with A4 Th11, N0 injury regarding AOSpine classification; (**b**) pedicel hypoplasia Th12; (**c**) surgical site with cantilevers connected to the pedicle screws which where employed for reduction maneuvers without using a rod; (**e**,**f**) reduction fixed with cantilevers ∂ Cobbs angle of 22°; (**d**) angle stable fixation of the rods to the pedicle screws using a glimbal-type technique; (**g**) a.p. view of definite instrumentation; (**h**) lateral view with additional VBR and lateral plating.

Anterior–posterior surgery can be performed in several combinations. Besides the named posterior options, anterior stabilization may either be monosegmental or bisegmental with anterior corpectomy, with or without decompression of the anterior column, and several options of reconstruction using autologous or allogeneic grafts, or static or expandable cage systems (Figure 1a–h). Additional anterior fixation with plate and screw constructs can be applied (Figure 2a–h) [48]. Expandable vertebral body replacement devices are typically used bisegmentally and, with exceptions, monosegmentally for anterior column reconstruction [49]. Iliac crest autologous bone grafts may also be used for anterior fusion, but have been reported to have high donor-side morbidity [36,44,50,51]. Faster fusion has been reported after additional anterior plate fixation in antero-posteriorly stabilized thoracolumbar fractures; however, without advantages regarding reduction loss or cage subsidence [52,53].

Combined anterior–posterior stabilization with expandable titanium cages emerged as a safe procedure with satisfactory radiological and clinical long-term outcomes, high fusion rates, and only minor loss of correction, still bearing all risks and complications prone to thoracotomy [52]. To decrease the invasiveness of the anterior approach, thoracoscopic and minimally invasive lateral approaches have been developed and widely accepted with satisfying results [54]. Continuing pain has been reported in patients having had anterior–posterior stabilization independently of open or minimally invasive anterior surgery [54].

## 6. Discussion

The surgical management of thoracolumbar spine fractures has advanced significantly over the last 25 years, driven by technological innovations, better understanding of spinal biomechanics, and a shift towards minimally invasive surgical approaches.

Refinements in pedicle screw and rod constructs with enhanced pull-out strength, modular and individualized constructs based on fracture patterns enabled a new era of construct stability. Polyaxial, cannulated, and fenestrated screws allow surgeons to exhaust their full potential together with minimally invasive or even percutaneous techniques. Over the last 25 years, spinal surgery has become less invasive, more precise, more personalized, with an improvement of intraoperative imaging, navigation and instrumentations leading to better clinical outcomes. There is still an ongoing debate of certain treatment strategies especially in incomplete and complete burst fractures of the thoracolumbar junction. The technical progresses with improved implants, instruments and retractor systems have led to an increase of minimal invasive procedures. Especially the posterior percutaneous procedures with the possibility to restore spinal alignment changed fractures treatment significantly in the last decades. In addition, the decrease of invasiveness of the anterior approaches changed the treatment strategies towards supporting the anterior column with hyperlordotic cages or vertebral body replacements in kyphotic burst fractures to restore and secure the sagittal alignment.

## 7. Conclusions

Combining surgical achievements with recent advancements intraoperative imaging, navigation, robotics, etc., spinal surgery will continue to change. Over the next 25 years, we are about to see robotics, AI, customized implants, regenerative therapies, digital twins, and much broader access to these advanced methods in spinal surgery in order to achieve the goal of safer surgery, faster recovery, more tailored treatment with less long-term adverse effects.

## Data Availability

No new data were created or analyzed in this study.
